# Supporting transplant athletes: perspectives on delivery of a sports performance and well-being service at the British Transplant Games

**DOI:** 10.3389/fspor.2024.1416896

**Published:** 2024-07-04

**Authors:** Elaine Duncan, Rachele Nateri, Abigail Lind, Sheila Leddington-Wright, Alison Bloxham, Lindsey Moffitt, David Sykes

**Affiliations:** ^1^ Department of Psychology, School of Health and Life Sciences, Glasgow Caledonian University, Glasgow, United Kingdom; ^2^ School of Life Sciences, University of Coventry, Coventry, United Kingdom; ^3^ AB Therapy Services, Therapy Lead, Transplant Sport UK, Devon, United Kingdom; ^4^ Glasgow International College, University of Glasgow, Glasgow, United Kingdom

**Keywords:** transplant games, transplant athletes, sport performance service-delivery, physical activity, well-being, reflective practice, trainee development

## Abstract

Evidence suggests that engaging in physical activity improves the mental and physical health of transplant recipients. An opportunity to be more active could be participating in the national and international network of Transplant Games. Although the literature on motivations for and the experience of taking part in the Games is available, little is known about what role applied practitioners, specifically sport and exercise psychologists could play as transplant recipients prepare and compete. This paper offers perspectives on the provision of a sports performance well-being service delivered at the British Transplant Games. The paper consists of several sections. The first offers background and how the service came into being. The second provides details of the model and philosophy that underpinned the service delivery. The third includes the trainee and exercise practitioner's casework and the challenges therein. Informed by the team's reflections and post-games survey the final section proposes recommendations for future applied sport and exercise services at this unique event.

## 1 Introduction

### 1.1 The transplant games and the transplant athlete

Solid organ transplant surgery (e.g., heart, kidney, lung, liver, pancreas) and hematopoietic cell transplants (e.g., bone marrow) are life-saving surgical procedures (1, 2). Recent research (3–5) and clinical guidelines (6) suggest that returning to an active lifestyle post-transplant may have a positive impact on the health and quality of life of transplant recipients (7, 8). An opportunity to be more active could be participation in the national and international network of Transplant Games. The Transplant Games, founded by Maurice Slapak, uses sports participation to raise awareness of organ donation and promote active lifestyles. The first Games (1978) were held in Portsmouth with only 99 athletes, from 5 countries and was a 1-day event. Now a network of national and international games exists with the potential to attract around 2,000 competitors, from 60 countries to week-long events. The Games is an individual and team multi-sports event including track and field, golf, swimming, cycling, racquet sports, volleyball, and more. Participants compete in age categories (e.g., under five, junior, 20–29, 30–39 through to 80–89). Transplant recipients who compete as Athletes (TxA) can be part of a larger team and affiliated with the hospital from which they received their transplant. The overall climate of the Games is one of participation, fellowship, survivorship (9) and inclusivity (10). Yet, research suggests that TxA display varying levels of ability from those who simply wish to take part to those who commit time to training and resources to achieve personal bests and world record performances (11). In addition to individual differences in performance capacity transplantation can cause significant physical, psychological, social, and emotional issues impacting upon general health status and may limit such participation (12–15). Studies of TxA motivations for and the impact of competing at the Games (11, 16–18) are evident, however apart from informal performance support during training camps and at the World Transplant Games in 2019 and 2023^1^ there is no extant literature on the role applied sport psychology had played in such a context (19). The authors believe this to be the first paper to offer perspectives on the delivery of a sports performance and well-being service at a British Transplant Games (BTG). Initially, this was offered as a pilot service with hopes of developing it further at future Games.

### 1.2 How the service provision came into being

The lead author, (ED), is an applied sport and exercise psychologist, counselor, and tutor within a Sport and Exercise Psychology taught doctorate program at Glasgow Caledonian University (GCU). For over a decade she has attended the BTG supporting her partner, a transplant athlete (author, DS). Such up-close and personal experience fueled research on transplant recipient well-being (with author SLW^2^) and the desire to offer sports psychology services to transplant games competitors. A mechanism for such could be recruiting trainee sport and exercise psychologists from the aforementioned taught doctorate with ED providing supervision of practice and insights from her BTG experience. This model of training would help GCU trainees meet program requirements including 450 client practice hours in diverse placements. Moreover, preparing trainees to function in complex and challenging environments necessitates exposure to real-life and dynamic practice environments (20, 21).

Collaboration with SLW (Sports Therapist) raised awareness of trainee placement opportunities at the BTG. Expanding from informal support offered by one physiotherapist at the 1999 BTG a Therapy Team was officially created in 2014. Over time the Therapy Team has consisted of trainees (on average 20) from diverse professions, (Physiotherapy, Sports Therapists, Sports Rehabilitators, Sports Masseurs, and Chiropractors) who gain hands-on experience treating competitors at the BTG and at the international transplant games circuit under the tutelage of qualified practitioners. The overall management of the Therapy Team is the remit of author AB (Physiotherapist). Discussions about incorporating a psychological service delivery were proposed in 2022 which led to this pilot at the BTG, Coventry in 2023.

Several GCU doctoral trainees responded to an expression of interest call. Two were selected (authors, RN and AL) based on their placement experience at live sporting events, implementing domain-specific psychological skills and training in psychological therapies. At the selection point, trainees had amassed over 550 client consultancy hours.

## 2 Nature of service delivery

### 2.1 Model and components of service delivery

Although models of delivery and evaluation of applied sport psychology exist (22, 23) and evaluation of the impact of applied sports psychology on performance exist (24) the realities of being a TxA and the ethos of BTG make a systemic approach to both difficult. TxA are typically not supported by coaches or other sports performance personnel. Moreover, competitors can register to play a sport they have never played before. Health vulnerabilities of transplant recipients (11) can mean interruptions to physical activity levels and competition (8); making it difficult to measure performance across time. Constraints aside, the goal was to offer a flexible service delivery model tailored to this client group's needs.

The service was designed to have three strands; psychological and emotional support; psychoeducation on physical activity and lifestyle factors; and psychological skills training (PST) for performance-related issues. The team adopted a transplant athlete-centered philosophy that considered the whole person (25, 26) and the wider context of participation at the BTG. In the context of transplant recipients, it is arguably even more important to adopt a holistic approach that places the well-being of the individual first and the performer second (27). As part of this philosophy, the therapeutic relationship remained central even within single-session consultations (28, 29). Adopting an empathic, non-judgmental manner (30–32), to develop trust and rapport (33) that prioritized hearing competitors' stories and a collaborative approach to interventions. A cognitive-behavioral theoretical framework underpinned the PST interventions (34) which have been effectively used with athletes (35), and evidence also supports cognitive-behavioral-based PST as an effective method to enhance psychological skills, behavior, and performance (36). A specific *in situ* example was the use of self-talk during competition breaks. Initially, not seeing self-talk as pertinent psychoeducation around this concept raised awareness of a client's undirected self-talk (37, 38). The client was surprised by the negative valence of their self-talk, but through collaboration the client and AL were able to brainstorm alternative, more adaptive self-talk that could be motivational and instructional, thus aligning better with that individual's performance (39).

Several factors determined which sports the psychology team supported. Practitioners' preferences and prior experience of sport(s) and that some sports were more conducive to pitch-side interventions whilst others were more accessible, for example, all racquet sports were held in the same venue. Refer to Table 1 below for a complete breakdown of the number & duration of sessions, the client type, and the nature of the client work.

**Table 1:** Nature of client work.

Sport attended	With^a^	Nature of client work^b^	Total hours
Athletics	1 TxA	IR, ME	1.0
Table tennis & badminton	1 TxA	PER, PST	1.5
Golf	1 TxA	SPA	1.0
Cycling	1 TxA	IS, RD	1.0
**Total 1-2-1 clients^c^**	**4 clients**		
Squash	4 TxA	TH, PER, DDG, ET, PST	2.0
Table tennis	3 TxA, 1 PD	SPA, TH, PER, TNC, DDG	2.0
Badminton	3 TxA	SPA, PER, DDG	1.5
Tennis	4 TxA, 1 P, 1 D	TH, PER, GR, TNC	3.3
Archery	3 TxA	IR, SPA, PST	2.0
Volleyball	1 TxA	TNC	1.0
Cycling	4 TxA	ME, PD, FPG	3.0
**Total pitch side clients^c^**	**25 clients**		
**Total number of clients**	**29 clients**	**Total client hours**	**19**.**3**

^a^ With: TxA, transplant recipient athlete; P, parent; D, donor (or combination).

^b^ Client work: IR, injury recovery; ME, management of (performance) expectations; TH, transplant history; PER, performance emotion regulation (pre and in-game); PST, psychological skills training; DDG, difficulties during (or) of the game/sport; SPA, sport psychology and its application for games; GR, game routines/visualization (pre and in-game); ET, experiences in therapy; TNC, honoring & being part of the transplant network and community; IS, imposter syndrome; RD, relationship difficulties; PD, performance debrief; FPG, future performance goals.

^c^ 1-2-1 or pitch side client service: 1-2-1 client contact was more intensive therapeutic sessions, akin to a counseling model, lasting from 30 min to 1 h (delivered before or during the BTG). The pitch side client contact was light-touch, brief intervention work at the point of play and could last anything from 5 min to 1 h but were typically 30 min.

### 2.2 Relationship to therapy team

The psychology service was incorporated under the Therapy Team in name only. For this pilot, the psychology team operated independently and differed in terms of trainee support level. Whilst other team trainees were directly supervised by SLW, and AB, the sport and exercise trainees worked independently of ED. This ensured client confidentiality as per professional practice guidelines (40, 41) and was consistent with placement experiences within the GCU program. To support practice, the psychology trainees engaged in one-to-one supervision with ED, typical of the doctorate program. A client referral system between the Therapy Team and the psychology service was not a feature of this pilot. However, AB did use clinical judgment to sign-post two clients to the service. ED produced a report on the use of psychology service which AB's include in the overall report on Therapy Team activities. This was shared with the event organisers, (Multi-Level Services, MLS) and the chairman of Transplant Sport, enabling resource planning at future events.

### 2.3 Raising awareness of service delivery

Awareness of the service was raised via two methods, firstly using relevant transplant community social media and secondly requesting that transplant Team Managers cascade our service advert to their team members. Interested TxA shared an initial online session with ED before onward referral to trainees to discuss pre-and/or during the Games support. At the event, the psychology team wore the official Therapy Team t-shirt and displayed accreditation passes to access all areas.

## 3 Reflections and challenges during service delivery

### 3.1 Initiating therapeutic relationships and finding confidential spaces

One key element was setting the scene for competitors; that is, succinctly outlining the nature of the psychological service offered and the overlap between physical activity, psychology, and behavior. This required skill in gauging the level of interest and degree of engagement in the service. Understanding what the client may seek from our interaction can be an important first step (42). Our brief contact interventions were guided by the following principles: prioritizing present psychological experience, observance of an athlete's verbal and non-verbal cues, and listening for entry points (43). These were important guides in initiating therapeutic encounters and part of the team's decision matrix of when to move through to more intensive and longer one-on-one sessions to supplement pitch-side style encounters (44). Brief interventions can be an effective mechanism for improving athlete psychological well-being (45) and adaptations can be made by sports psychologists to work effectively within this time-limited environment (46). Related to this is the challenge of working at live sporting events whilst preserving confidentiality. The event's dynamic nature meant it was often not possible to find a quiet, confidential space as noted by other practitioners at live events (47, 48). It was, therefore, important to ensure that attendees felt comfortable talking in the space we had available, a challenge evidenced by other service delivery teams at live sporting events (49).

### 3.2 Managing the climate and emotional labor of the games

At one extreme the climate was one of joy, hope, and celebration of life: yet at the other, a complex interplay between gratitude and guilt. Competitors spoke of the pressure they put themselves under, of performing in front of the donors' families, to somehow make their loss more bearable, and to give back. Tebbens (50) has highlighted this phenomenon that others call “survivor guilt” (51). Author (RN) bore witness to this emotional dichotomy when transplant recipients expressed gratitude towards their donors and donors’ families, across breakfast with an athlete and their family, and when watching a tennis as a parent disclosed the story of their donor child. The personal wants to simply meet another human in suffering but the professional may need to draw upon emotion-regulation skills to hold onto boundaries that support a practitioner's well-being (52). These experiences of blurred boundaries between personal and professional roles have been reported by others providing services at live sporting events (53–55).

Part of the Transplant Games experience is the opportunity to attend the opening ceremony. A street parade of teams, families, and friends, akin to the Olympics, albeit scaled down in duration, and budget. The psychology team chose to attend this ceremony sensing the value of an immersive approach to consultancy that advocates familiarization with the dynamics of a live sports event, or tournament (56). The author RN reflected on how overwhelming and emotional this was, hitting them most unexpectedly when a poem was recited by a grateful sister for having been given the chance to grow up with her little brother thanks to a lifesaving donation only made possible by another human being losing their own life. It is possible that trainee preparation for and confidence in managing in-session client work may not necessarily consider what is needed in terms of coping with the broader context of a sporting event like the Games. Hings and colleagues (52) have looked at the demands of the applied practice of sport and exercise psychologists in training identifying what they called, an emotional experiential gap. This gap is defined as inexperience in managing emotional labor and can be reduced with appropriate support, education, and experience to better prepare them for the challenges they face (52). Emotional labor is defined as the management and verbal and non-verbal expression of emotions, in line with the requirements of a specific situation (57). Studies have found that in the healthcare professions, emotional labor demands are often difficult to cope with (58–60). Studies looking at the emotional labor of coaches and support staff at the Olympic Games (61) have also shown that most of the participants described the sports event as an emotional roller coaster with feelings of excitement, low mood, and emotional exhaustion. These results are also consistent with Hayton's (62) on volunteers taking part in a sport-based project. Hings and colleagues' results (52) have shown that support can make a difference in trainees' applied practice. As a team, we quickly recognized the emotional labor of the Games and the importance of having peer supervision in place to better manage the emotional demands of the event and support each other (63).

## 4 Key recommendations for service delivery at future transplant games

Evaluation and reflection-in-action are necessary components of applied sports psychology practice (64–66). Although the response rate to the post-games survey wasn't high (*n* = 19) author LM was still able to extract key themes which alongside the psychology team's reflection shaped recommendations for future service delivery.

Half of the respondents who had prior knowledge of the service found it difficult to access, suggesting that the psychology team liaised with the event organizers to improve signage to the service at the BTG. Placing signage in areas known to have higher footfall (e.g., the multi-event sports venues) and stationing members of the psychology team in the hubs where the other Therapy Team practitioners work could improve this. A more structured process of onward referral from practitioners within the Therapy Team to the psychology service is also proposed. This would require the creation of guidelines to help Therapy Team practitioners judge when physical and restorative support needs to be supplemented with psychological support.

In answer to the survey question “What role could sport, and exercise psychologists play at future transplant games” several competitors said they would value help managing the nature of competing at the games with one saying they “never produce good throws under the pressure of the games”. Others reported that psychologists could help with “self-doubt” and “managing stress and anxiety”. Several mentioned how psychologists could help competitors “understand their feelings” about being a transplant recipient and help with “personal development”. There was, therefore, some synergy between these priorities and the psychology team's applied work. However, a larger study of TxA needs before the BTG could better inform future service delivery, a strategy adopted by a psychology service delivery team in preparation for 2024 Olympic games (67).

The psychology trainees recommend enhancing pre-games preparation, specifically, more education on the psychological and physical milieu of being a transplant recipient. This should include more information on the impact of a transplant recipient's medication regime and factors associated with managing a long-term health condition. The addition of a blanket consent process to access the service could be alongside the Game's registration process. This may be a more agile and fit-for-purpose approach to informed consent and raise awareness of the service earlier. The psychology team should also adopt online and/or telephone modes for supervision and debriefing, this may have eased the logistical difficulties the team experienced attempting to conduct in-person.

Moving beyond this pilot would necessitate a more systematic and multi-dimensional approach to service evaluation. Measures proximal to service delivery may be preferable to a post-games approach. Assessing client's understanding of the interventions they engaged in, and outcomes such as well-being and performance goals could be incorporated. We could also evaluate the practitioner's skills, such as empathy, and degree of fit to client needs (68).

## 5 Conclusion

This paper presented perspectives from the first integrated sports performance and well-being service delivered at the British Transplant Games. It was feasible to do so and the responses to the post-games survey suggest a sports psychology service would be of value to TxA. This experience provided trainee sport and exercise psychologists with an opportunity to work with a broader base of client presentations than typically exposed to with much to reflect upon, not least how to navigate the climate and emotional labor of the Games. Our recommendations are essential if ambitions to upscale the sports performance and well-being service at future Games are to be realized.

## Data Availability

The raw data supporting the conclusions of this article will be made available by the authors, without undue reservation.

## Footnotes

^1^In an email from L. O'Halloran, PhD (lisa.o'halloran@ntu.ac.uk) in April 2024.

^2^Findlay SE. *Wellbeing in Solid Organ Transplant Recipients*. Glasgow: GCU (2020).
